# Supplementation with *Agrobacterium* sp. FN01-Derived Crude Product Dominated by L-β-Galactoglucan Enhances Growth Performance and Nutrient Utilization and Modulates Intestinal Microbiota in Pigs

**DOI:** 10.3390/ani16172752

**Published:** 2026-09-02

**Authors:** Mengli Chen, Zicheng Zhang, Lingling Du, Jin’e Yu, Huiming Wang, Xiaodan Yu, Siyang Zhang, Li’er Lin, Cimin Long, Pan Huang, Xiangfeng Kong, Xiaoxiao Liang, Jianzhong Li, Xia Xiong, Yulong Yin

**Affiliations:** 1 Hunan Provincial Key Laboratory of Animal Intestinal Function and Regulation, Hunan International Joint Laboratory of Animal Intestinal Ecology and Health, Laboratory of Animal Nutrition and Human Health, College of Life Sciences, Hunan Normal University, Changsha 410081, China; 2 Hunan Provincial Key Laboratory of Animal Nutritional Physiology and Metabolic Process, National Engineering Laboratory for Pollution Control and Waste Utilization in Livestock and Poultry Production, Institute of Subtropical Agriculture, Chinese Academy of Sciences, Changsha 410125, China; 3 Chengdu Sydix Biotech Co., Ltd., Chengdu 610041, China; 4 Hunan Liushahe Huazhu Ecological Animal Husbandry Co., Ltd., Changsha 410600, China; 5 College of Advanced Agricultural Sciences, University of Chinese Academy of Sciences, Beijing 100049, China; 6 College of Bioengineering, Henan University of Technology, Lianhua Street, Zhengzhou 450001, China

**Keywords:** L-β-galactoglucan, pigs, growth performance, gut microbiota

## Abstract

Polysaccharides are well known for their immunomodulatory properties and gut microbiota-regulating functions; however, their digestive stability and structure-dependent biological mechanisms remain poorly understood. This study investigated the in vitro enzymatic resistance and intestinal regulatory effects of a novel L-β-galactoglucan (Mw 400–520 kDa) derived from *Agrobacterium* sp. FN01, and further evaluated the in vivo effects of its corresponding crude fermentation product on growth performance and intestinal metabolism in pigs. Twenty-seven-day-old weaned piglets were randomly allocated to three treatments, with 16 pens per treatment and 13 piglets per pen, and fed basal diets supplemented with 0, 200, or 400 mg/kg of the crude product. The results showed that L-β-galactoglucan exhibited strong resistance to pancreatic α-amylase and glucoamylase under the tested in vitro conditions, with minimal release of low-molecular-weight oligosaccharides during hydrolysis. The 400 mg/kg crude product supplementation significantly improved growth performance, reduced the feed-to-gain ratio, and increased the apparent total tract digestibility of dry matter, crude protein, crude fat, and gross energy (*p* < 0.05). In addition, dietary supplementation with this crude product increased intestinal microbial diversity, enriched *Halalkalibacter urbisdiaboli*, and increased colonic concentrations of isobutyrate and isovalerate (*p* < 0.05). Functional metagenomic analysis further revealed enhanced microbial carbohydrate metabolism and energy metabolic pathways. In conclusion, under the in vitro conditions tested in this study, the novel L-β-galactoglucan exhibited resistance to the two tested enzymes. In the pig trial, this polysaccharide improved growth performance and nutrient utilization, and modulated the intestinal microbial composition. These observations suggest that the fermentation product may be promising for animal feeding applications.

## Simple Summary

Polysaccharides play important roles in regulating animal immunity and intestinal health, while their digestive stability and in vivo regulatory effects remain insufficiently clarified. In this study, we characterized a novel water-soluble L-β-galactoglucan isolated from fermented products of soil-originated *Agrobacterium* sp. FN01 using purified preparations in vitro, and further evaluated the in vivo effects of the corresponding L-β-galactoglucan-dominated crude fermentation product on growth performance, nutrient digestibility, intestinal microbiota, and short-chain fatty acid metabolism in pigs. Supplementation with this crude product improved nutrient utilization by increasing the apparent digestibility of dry matter, crude protein, and other nutrients, increasing average daily gain, and decreasing feed conversion ratio. It also reshaped intestinal microbial composition by improving gut microbial diversity and raising levels of colonic isobutyric acid and isovaleric acid, two typical branched-chain fatty acids produced by intestinal microbial fermentation. In vitro assays showed that L-β-galactoglucan was resistant to hydrolysis by pancreatic α-amylase and glucoamylase. Combined with the in vivo biological responses obtained from the crude product, this material exhibits promising potential as a natural feed additive for swine production.

## 1. Introduction

Given the increasingly severe concerns over antibiotic resistance and biosafety risks, natural polysaccharide bioactive molecules have attracted widespread attention in biofunctional molecule research and physiological regulation due to their excellent biocompatibility and multiple health-benefiting potentials [1,2]. Polysaccharides are widely present in algae, plants, fungi, and microbial metabolites, exhibiting multiple activities such as antioxidant, immunomodulatory, and antibacterial properties. However, their stability in the gastrointestinal environment, bioavailability, and interaction mechanisms with the gut microbiota, particularly how structural specificity influences their functional expression, remain key directions in current research [3].

Microbial polysaccharides refer to a class of functional high-molecular-weight polymers synthesized and secreted by microorganisms such as bacteria and fungi during their metabolic processes, typically characterized by high structural complexity and branching degrees. Compared to polysaccharides derived from plants or animals, microbial polysaccharides offer advantages such as diverse sources, high production efficiency, ease of large-scale fermentation, and resistance to seasonal or environmental fluctuations, making them a recent focus of research on dietary functional components [4]. β-glucans, as representative non-starch polysaccharides, are widely distributed in the cell walls of various biological sources such as yeast, bacteria, fungi, and grains. Their biological functions are closely related to their structural characteristics, including monosaccharide composition, glycosidic bond types, and branching patterns. Due to its unique β-1,3, β-1,4, and β-1,6 glycosidic bond structure, β-glucan has demonstrated immune-enhancing, anti-inflammatory, and intestinal barrier protective activities in various animal models [5,6,7]. The physiological activity of polysaccharides is highly correlated with their structure. Specifically, monosaccharide composition, glycosidic bond types, degree of branching, molecular weight distribution, and spatial conformation collectively determine their resistance to digestion in the upper gastrointestinal tract and the degree to which they are utilized by specific microbial fermentation pathways in the intestine, thereby influencing the profile of short-chain fatty acids and the structure of microbial communities [8].

Notably, galactoglucan, as a structurally distinct subclass of microbial polysaccharides (or more specifically, β-glucans), is characterized by the presence of galactose side chains attached to its backbone. This structural feature endows it with distinct physicochemical properties and physiological functions compared to typical linear or branched β-glucans. Given that these structural modifications are likely to influence its interactions with and modulation of the gut microbiota—a key mechanism for many polysaccharide bioactivities—investigating its specific effects is of particular interest. The core polysaccharide, L-β-galactoglucan targeted herein, has been fully structurally elucidated with established preparation protocols in previous research [9]. Although the strain fermentation batches differ, the consistent fermentation and purification workflow yields a polysaccharide sharing the characteristic backbone structure of reported L-β-galactoglucan. Therefore, this study selected L-β-galactoglucan derived from the fermentation products of *Agrobacterium* sp. FN01 isolated from highland soil. The molecular weight stability of this compound before and after in vitro digestion was analyzed using high-performance gel permeation chromatography–refractive index (HPSEC-RI). The L-β-galactoglucan-dominated crude fermentation product used for the in vivo feeding trial was obtained from a single production batch. Three independent production batches of purified L-β-galactoglucan were additionally prepared and subjected to HPSEC-RI analysis to assess batch-to-batch variation in molecular weight distribution. For the batch used in the pig feeding trial, the polysaccharide content was determined as 75.10% on a dry-matter basis, with residual components consisting of 1.72% moisture, 13.30% crude protein, and 9.5% ash. Notably, the purified L-β-galactoglucan for in vivo structural and enzymatic hydrolysis characterization was obtained by laboratory-scale isolation, and its yield was insufficient for large-scale pig feeding. Therefore, the industrial crude fermentation product described above was adopted for the in vivo trial. Additionally, animal experiments with growing pigs, metagenomic sequencing, and short-chain fatty acid detection were combined to systematically evaluate the effects of this crude fermentation product on intestinal microbiota and digestive metabolism. This study aimed to probe the potential mode of action of its major component, L-β-galactoglucan, and lay a theoretical foundation for its basic biological research and swine feed application.

## 2. Materials and Methods

### 2.1. Animal Ethics

The experimental design and procedures used in this study were approved by the Animal Care and Use Committee of the Institute of Subtropical Agriculture, Chinese Academy of Sciences (No. ISA-2024-00-16, approval date: 23 May 2024). All animal experiments were performed in accordance with the ARRIVE guidelines and relevant national laboratory animal welfare regulations.

### 2.2. Preparation and Basic Characterization of L-β-Galactoglucan

L-β-galactoglucan was produced by fermentation of *Agrobacterium* sp. FN01 following a patented procedure. Briefly, the strain was cultivated in a liquid fermentation medium under controlled conditions, after which bacterial cells were removed by centrifugation, and the extracellular polysaccharide was recovered from the supernatant. The crude polysaccharide was then concentrated and subjected to ethanol precipitation, followed by deproteinization using a modified Sevag method. The resulting polysaccharide fraction was desalted, spray-dried, and supplied as a powder. The polysaccharide was a high-molecular-weight, branched galactoglucan mainly composed of glucose and galactose residues [9].

### 2.3. HPSEC-RI for Molecular Weight Analysis

The molecular weight distribution of L-β-galactoglucan and its enzymatic hydrolysis products were analyzed using high-performance gel permeation chromatography–refractive index detection (HPSEC-RI). Digestion was performed according to AOAC 2017.16, involving sequential enzymatic hydrolysis using pancreatic α-amylase followed by amyloglucosidase to simulate the enzymatic environment of the small intestine. The digestion solution concentration was approximately 2.5 g/L (20 mg dissolved in 8 mL solution). After digestion, the samples were heated at 100 °C for 10 min to inactivate enzymes and microorganisms, diluted threefold, and filtered through a membrane filter for later use. Chromatographic separation was performed using three series-connected columns (including a guard column) of the Tosoh Bioscience TSKgel Super AW series (Tosoh Bioscience, Tokyo, Japan), with 0.4 M NaNO_3_ (pH 2.5, adjusted with nitric acid) as the mobile phase, and elution was carried out at 55 °C. The detector was a differential refractive index detector. Molecular weight calibration was performed using Pullulan series standards with known molecular weights and disaccharides (342 Da).

### 2.4. Animals and Experimental Design

The doses of crude L-β-galactoglucan used in the formal experiment were selected based on a preliminary 42 d feeding trial (Supplementary Table S1), in which 28-day-old weaned piglets (Duroc × Landrace × Large White; 7.47 ± 0.23 kg) were fed diets supplemented with 0, 200, 400, or 600 mg/kg of crude L-β-galactoglucan. Based on the results of this preliminary study, 0, 200, and 400 mg/kg were selected for the subsequent experiment.

This experiment used 624 piglets (Duroc × Berkshire × Ningxiang pigs), weaned at 27 days of age with an initial body weight (BW) of 6.89 ± 1.38 kg. Approximately half of the pigs were castrated males, and the remaining were intact females. At 37 days of age, stratified randomization was conducted on pens rather than individual pigs. All pens were stratified based on the average initial body weight and sex ratio within each pen, and each whole pen was randomly assigned to one of the three treatments: 0 mg/kg (control group), 200 mg/kg, and 400 mg/kg crude L-β-galactoglucan in the diet. Each treatment included 16 replicate pens, and each pen housed 13 piglets with a balanced sex ratio at the beginning of the trial.

The feeding program included two phases: a nursery phase (37–65 days of age) and a finishing phase thereafter (66–196 days of age). The nursery and finishing diets used in this experiment were commercial feeds supplied by Anyou Biotechnology Group Co., Ltd. (Jiangyin, China), and their ingredient composition and nutrient levels are presented in Table 1. All diets were formulated to meet the nutrient requirements recommended by NRC (2012) [10]. Pigs were weighed under fasting conditions on the morning of D37, D66, D96, D153, and D197. Feeding phases were defined as D37–65 (29 d), D66–96 (31 d), D97–153 (57 d), and D154–196 (43 d), with a total feeding duration of 160 d, which corresponded to the start of the trial, nursery phase, early finishing phase, mid-finishing phase, and late finishing phase. The pen was the experimental unit for BW, ADG, ADFI, and F/G because feed intake was recorded per pen. The calculation formulas for growth performance indicators were as follows: ADG = (final phase average BW − initial phase average BW)/phase days; ADFI = total phase feed intake/total animal-days; and F/G = ADFI/ADG. Dead or removed animals were excluded when computing pen-level average body weights, while the pen was still kept as an experimental replicate. Animal-day adjustment was only applied for ADFI calculation to account for mortality or animal removal. Total feed consumption included only feed intake corresponding to the survival period of animals that died or were removed during the experimental period. No animal-day adjustment was performed for ADG calculation.

During the entire experimental period, the indoor temperature was maintained at 24–28 °C and relative humidity was controlled at 55–70%, and the pigs had ad libitum access to feed and water. Continuous mechanical ventilation was operated to ensure air quality inside the facility. Environmental management, including regular cleaning, disinfection, and control of temperature and humidity, was performed in accordance with standard farm protocols to maintain a dry and hygienic environment. No therapeutic antibiotics were administered during the feeding period. Other feeding management procedures followed routine protocols. Daily health monitoring was carried out throughout the trial. At day 66, pigs with body weight below 15 kg were removed from their pens according to the pre-defined trial criteria, as excessively low body weight would confound the evaluation of finishing-phase performance. These animals were included in the growth-performance calculation for the D37–66 period, but excluded from all finishing-phase indices (D66–196). Several pigs died or suffered severe illness during the experiment. These individual animals were excluded from growth-performance calculations, but no entire pen was excluded from statistical analysis. Mortality and animal-removal information for each pen is summarized in Supplementary Table S2. At the end of the experiment, selected pigs were rendered unconscious via electrical stunning followed by exsanguination in accordance with standard commercial humane slaughter procedures.

### 2.5. Sample Collection

For digestibility, SCFA and metagenomic analyses, 10 pigs per treatment were selected from 10 separate pens, with one pig sampled from each pen. Three days before slaughter, fresh feces were collected every morning for three consecutive days by gently stimulating the rectum with sterile cotton swabs to induce defecation, avoiding contamination by urine and feed residues. All fresh fecal samples were placed into self-sealing bags and immediately stored at −20 °C after collection. Before nutrient determination, fecal samples collected over three days from the same pig were fully mixed for subsequent analysis. Meanwhile, approximately 200 g of diet samples per group were collected by the quartering method and stored at 4 °C. At the end of the trial, another 10 pigs per group were selected to collect colonic digesta. The samples were placed into sterile centrifuge tubes and preserved at −80 °C for subsequent metagenomic sequencing. All samples collected from each group were used for the determination of apparent digestibility. Eight samples per treatment were randomly selected for metagenomic sequencing and functional analysis. From these eight sequenced samples, six were further randomly subsampled for SCFA. Complete sample mapping information is provided in Supplementary Table S4.

### 2.6. Apparent Digestibility

Diets and fecal samples were ground to pass through a 40-mesh screen. Fecal samples were dried at 65 °C and stored for subsequent analysis. Acid-insoluble ash (AIA) was used as an indicator to determine the apparent digestibility of dry matter (DM), gross energy (GE), crude protein (CP), and crude fat (EE) in the diet and feces. Dry matter was determined according to GB/T 6435-2014 [11], and AIA was analyzed following GB/T 23742-2009 [12]. Crude protein was measured using the Dumas combustion method, and nitrogen content was converted to CP using a factor of 6.25. Ether extract was determined by Soxhlet extraction using petroleum ether as the solvent. Gross energy was measured using an adiabatic bomb calorimeter. All analytical procedures were conducted in accordance with Chinese national standard methods. The formula for calculating the apparent digestibility of nutrients was: Apparent digestibility (%) = [1 − (A1 × F2)/(A2 × F1)] × 100, where A1 and A2 represent the AIA content (%) in the diet and fecal samples, respectively; F1 and F2 represent the nutrient content (%) in the diet and fecal samples, respectively.

### 2.7. DNA Extraction and Sequencing

Metagenomic DNA was extracted from colonic digesta samples using the DNeasy^®^ PowerSoil^®^ kit (Qiagen, Hilden, Germany). Approximately 1 μg of genomic DNA was fragmented to ~350 bp by Covaris sonication. Libraries were constructed following the NEBNext^®^ Ultra™ DNA Library Preparation Kit protocol, including end repair, A-tailing, adapter ligation, purification, and PCR amplification. Library quality and insert size were verified by AATI, and effective library concentration was quantified via qPCR (only libraries >3 nM were qualified). Qualified libraries were pooled and sequenced on the Illumina NovaSeq platform with paired-end 150 bp (PE150) at Novogene Co., Ltd. (Beijing, China).

### 2.8. Metagenomic Data Analysis

Raw reads were preprocessed with fastp (v0.23.1) to obtain clean reads. Reads containing adapters, excessive low-quality bases (Q ≤ 5 over 50% of the length), or >10% ambiguous N bases were discarded. Bowtie2 (v2.5.4), was used to filter host-origin reads against the pig reference genome Sus scrofa11.1 (GCA_000003025.6) with parameters --end-to-end, --sensitive, -I 200, -X 400. Clean non-host reads were assembled using MEGAHIT (--presets meta-large, v1.2.9). Scaffolds were split at N gaps to generate scaftigs. ORF prediction on scaftigs (≥500 bp) was performed by MetaGeneMark.hmm (v2.1); sequences shorter than 100 nt were removed. Predicted genes were clustered by CD-HIT (v4.5.8) (-c 0.95, -aS 0.9, -G 0, -g 1, -d 0) to build a non-redundant gene catalog. Bowtie2 was applied to map clean reads to the gene catalogue, and genes with ≤2 mapped reads were filtered out. Gene abundance was calculated based on mapped read counts and gene length. For annotation, unigenes were aligned using DIAMOND (v2.1.9) with the parameters “blastp, -e 1 × 10^−5^”. Taxonomic classification was conducted against Micro NR (sequences extracted from NCBI NR database, https://www.ncbi.nlm.nih.gov/, accessed 5 January 2025) using the LCA algorithm. Functional annotation was performed against KEGG, eggNOG v5.0, CAZy, VFDB and PHI databases. Database information: NCBI NR (downloaded: 10 January 2025), KEGG database (downloaded: 10 January 2025), and eggNOG v5.0 (downloaded: 10 January 2025). In community structure analysis, α-diversity indices (e.g., Shannon index, Chao 1 index) and β-diversity metrics (e.g., Bray–Curtis distance) were calculated at the phylum, genus, and species levels. The Bray–Curtis distance matrix was generated for principal coordinate analysis (PCoA), PERMANOVA, and PERMDISP, with 999 permutations applied for both permutation-based tests to evaluate intergroup differences in bacterial community structure. Differential taxa and functional genes (KO and eggNOG entries) were identified using the MetagenomeSeq (https://bioconductor.org/packages/metagenomeSeq/, accessed 10 January 2025) approach. Raw abundance data were normalized by cumulative sum scaling (CSS). Prior to differential abundance testing, taxonomic features (phylum, genus, and species level) and functional profiles (KO, eggNOG) were pre-filtered by prevalence. Only features present in at least 15% of all samples were retained for subsequent differential analysis. Hypothesis testing was performed to generate raw *p*-values, which were further adjusted via FDR correction to calculate *Q* values. All statistical outputs, including raw *p*-values, *Q*-values, effect-size estimates, and prevalence information, are summarized in Supplementary Table S6. Taxa and functional genes with *Q* < 0.05 were selected for visualization. 

### 2.9. Short-Chain Fatty Acid Analysis

Short-chain fatty acids (SCFAs) content in colonic digesta was determined using solid-phase extraction (SPE) combined with gas chromatography–flame ionization detection (GC-FID, Agilent Technologies, Santa Clara, CA, USA), following the method described by Zheng et al. [13]. SCFAs included acetate, propionate, isobutyrate, butyrate, isovalerate, valerate, and caproate.

The specific method is as follows: Weigh 50 mg of colonic digesta sample and place it in a 2 mL centrifuge tube. Add 400 µL of acetone, homogenize using a handheld tissue grinder, then add 600 µL of acetone, and vortex-mix thoroughly for 3 min. Centrifuge the sample at 6000× *g* at 4 °C for 10 min, and collect the supernatant for SPE. Activate the SPE column (Bond Elut Plexa, Agilent) with 1 mL of acetone, remove any residual solvent, and load the entire supernatant onto the column. Allow the sample to elute by gravity and collect the eluent in a clean centrifuge tube. The collected solution is directly used for GC-FID analysis. GC-FID analysis conditions: Chromatography column: DB-FFAP (30 m × 0.25 mm × 0.25 µm, Agilent); carrier gas: nitrogen; flow rate: 1 mL/min; injection port temperature: 280 °C; detector temperature: 250 °C; and hydrogen and air flow rates: 40 mL/min and 300 mL/min, respectively. The column temperature program was set as follows: initial 50 °C, held for 1 min, then increased at 15 °C/min to 120 °C, followed by an increase at 6 °C/min to 200 °C, and finally increased to 235 °C and held for 3 min for washing. The injection needle was cleaned with high-purity water and acetone before each injection. The concentrations of SCFAs were calculated using the external standard method based on the standard curve.

### 2.10. Statistical Analysis

For correlation analysis, both Spearman’s rank-order correlation and partial correlation (treatment group as a confounder) were performed using the psych package in R software (Version 4.5.1; R Foundation for Statistical Computing, Vienna, Austria). Raw *p*-values from Spearman and partial correlation were adjusted separately with the Benjamini–Hochberg FDR procedure to control multiple-testing risk, and the significance threshold was set at FDR-adjusted *Q* < 0.05. Correlation network diagrams were plotted using the online platform CNSknowall (available at: https://www.cnsknowall.com/, accessed on 20 August 2025).

Experimental data were analyzed using SPSS statistical software (Version 26.0; SPSS Inc., Chicago, IL, USA). The overall effects of Treatment, Time, and Treatment × Time interaction on repeatedly measured body weight were evaluated using a linear mixed model, with pen regarded as the random experimental unit. Other indicators were compared using one-way analysis of variance (ANOVA) and Tukey’s post hoc test. For datasets that failed to meet the homogeneity-of-variance and normality assumptions for parametric ANOVA, the Kruskal–Wallis H-test was applied. Differences were considered significant at *p* ≤ 0.05. A tendency toward difference was recognized when 0.05 < *p* ≤ 0.10, while no significant difference was observed at *p* > 0.05. The data are expressed as means and standard error of the mean (SEM).

## 3. Results

### 3.1. Molecular Weight Analysis Based on HPSEC-RI

**Figure 1:**
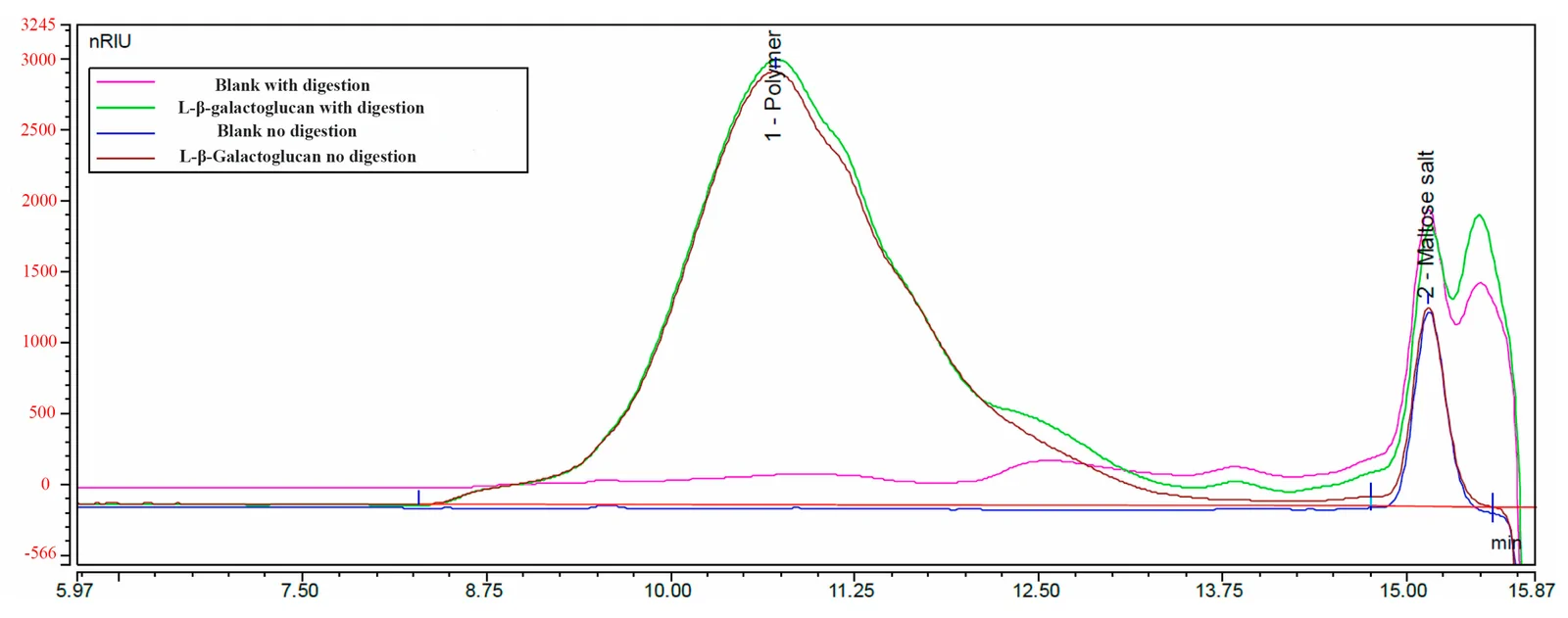
HPSEC-RI chromatogram of L-β-galactoglucan and blank controls under digestion and no digestion conditions. The chromatogram shows the relative abundance of L-β-galactoglucan and blank controls subjected to simulated digestion or not. The peaks represent the target polymer as well as a mixture of monosaccharides, disaccharides, and salt-like substances (labeled “2−Maltose salt”), with the baseline indicating the presence of small molecules or impurities. The *y*-axis shows the normalized relative units, and the *x*-axis represents the retention time in minutes. Blank with digestion: contained digestion buffer plus digestive enzymes, without L-β-galactoglucan substrate. Blank no digestion: contained only digestion buffer, with no enzymes and no L-β-galactoglucan substrate. Note: The label “2−Maltose salt” shown in the chromatogram is an auto-generated label from the instrument software. This peak corresponds to a mixture of monosaccharides, disaccharides, and salt-like substances. The red horizontal line indicates the baseline.

The molecular weight distribution and digestibility of L-β-galactoglucan were analyzed by chromatographic profiling before and after in vitro enzymatic digestion. Figure 1 presents the chromatograms of L-β-galactoglucan compared with the blank control group. A major polysaccharide peak was observed in the retention time range of 7–14 min, together with the high PDI (12–18), indicating a broad molecular weight distribution. After 14 min, a secondary increase in signal intensity was observed, corresponding to small-molecular-weight components such as monosaccharides, disaccharides, and salt-like substances. Table 2 shows the content of substances in digested and undigested samples. The results indicate that the main peak accounts for 87–88% of the mass in undigested samples, and the main peak still accounts for 85–86% after digestion. The slight reduction demonstrated that most structural parts remained intact during digestion. After digestion, the small-molecule peaks increased to 2–3%. These results suggest that L-β-galactoglucan has a molecular weight of 400–520 kDa and exhibits resistance to the tested enzymes under these in vitro conditions.

### 3.2. Effects of Crude L-β-Galactoglucan on Growth Performance of Wean-to-Finish Pigs

Initial BW did not differ among treatments (*p* = 1.000; Table 3). On d 96, there was a tendency for higher BW in the 400 mg/kg group compared with the control and 200 mg/kg groups (*p* = 0.095). On D153 and D197, pigs in the 400 mg/kg group had significantly greater BW than pigs in the control and 200 mg/kg treatments (*p* < 0.05). During the nursery phase (D37–65), pigs fed diets supplemented with 200 mg/kg had greater ADFI compared with the control and 400 mg/kg groups (*p* = 0.003). Meanwhile, the 400 mg/kg treatment achieved a lower F/G ratio during the nursery phase. In the growing-finishing period, no significant differences in ADFI and F/G were observed between supplemented groups and the control during D66–96. For the remaining periods, the 400 mg/kg group had significantly higher ADG accompanied by significantly lower F/G compared with the control (*p* < 0.05). Supplementation with 200 mg/kg and 400 mg/kg significantly reduced F/G during D154–196 and D66–196. In contrast, the 400 mg/kg group persistently improved body weight, ADG, and feed efficiency throughout the experiment, with prominent effects observed in the mid-to-late growing-finishing phase. The linear mixed model detected significant main effects of Treatment (*p* < 0.0001) and Time (*p* < 0.0001), as well as a significant Treatment × Time interaction (*p* < 0.0001) for body weight, demonstrating that the influence of crude L-β-galactoglucan on body weight varied across growth stages.

### 3.3. Effects of Crude L-β-Galactoglucan on Apparent Digestibility in Finishing Pigs

Compared with the control or 200 mg/kg group, pigs fed 400 mg/kg exhibited higher apparent digestibility of crude fat (*p* = 0.001). Supplementation with either the 200 mg/kg or 400 mg/kg group increased the apparent digestibility of dry matter, crude protein, and gross energy in finishing pigs (*p* = 0.001, Table 4).

### 3.4. Intestinal Microbial Community Analysis

#### 3.4.1. Effects of Crude L-β-Galactoglucan on Intestinal Microbial Diversity

**Figure 2:**
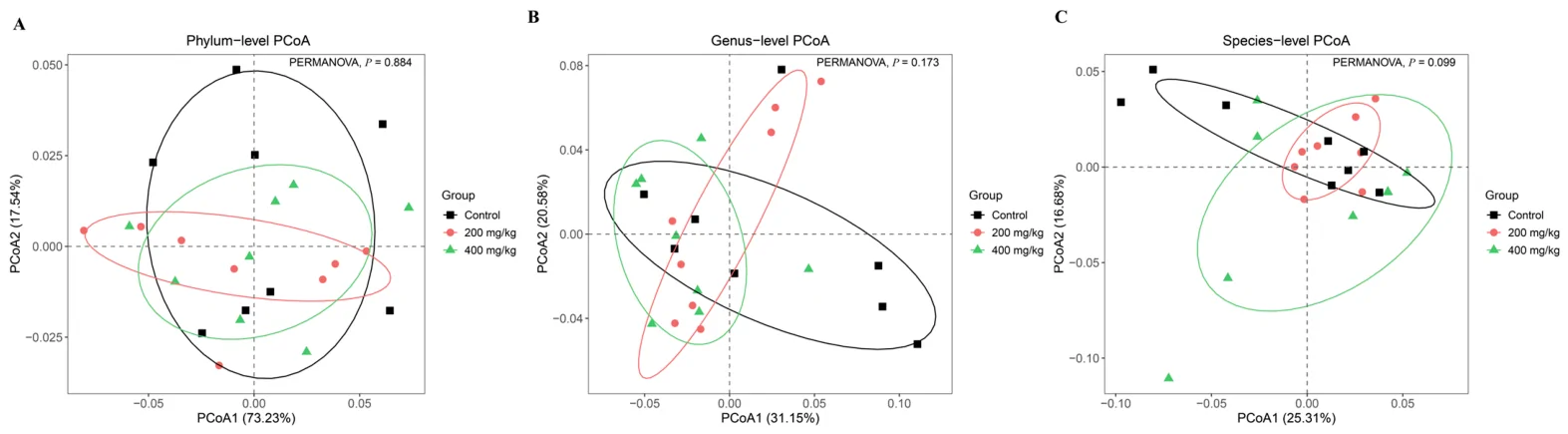
Effects of crude L-β-galactoglucan on the β-diversity of gut microbiota in colonic digesta of finishing pigs based on principal coordinate analysis (PCoA) (*n* = 8). (**A**) Phylum level; (**B**) genus level; (**C**) species level. Each point represents an individual sample, and ellipses indicate group clustering. The percentages explained by PCoA1 and PCoA2 are shown on the axes.

The effects of crude L-β-galactoglucan on gut microbial diversity in the pig colon microbiota were evaluated through metagenomic sequencing. α diversity analysis showed that the 200 mg/kg group significantly increased the Chao 1 index at the phylum level (*p* = 0.030, Table 5). This result demonstrated that low-dose supplementation could effectively improve the richness of intestinal microbial flora. A trend toward an increase in the ACE index was also observed at the phylum level (*p* = 0.057, Table 5). At the species level, the Simpson index tended to increase in both the 200 and 400 mg/kg groups relative to the control group (*p* = 0.096). These trends implied that this polysaccharide might help balance the distribution of intestinal microorganisms. PERMANOVA revealed no statistically significant differences in overall bacterial community structure among treatments across all taxonomic levels (Figure 2). PERMDISP confirmed the homogeneity of multivariate dispersion among groups at each taxonomic level (Supplementary Table S5). Collectively, these findings indicated that high-dose crude L-β-galactoglucan tended to modulate the overall composition of the intestinal microbiota, although these effects did not reach statistical significance.

#### 3.4.2. Effects of Crude L-β-Galactoglucan on Total Species and Dominant Microbial Communities

**Figure 3:**
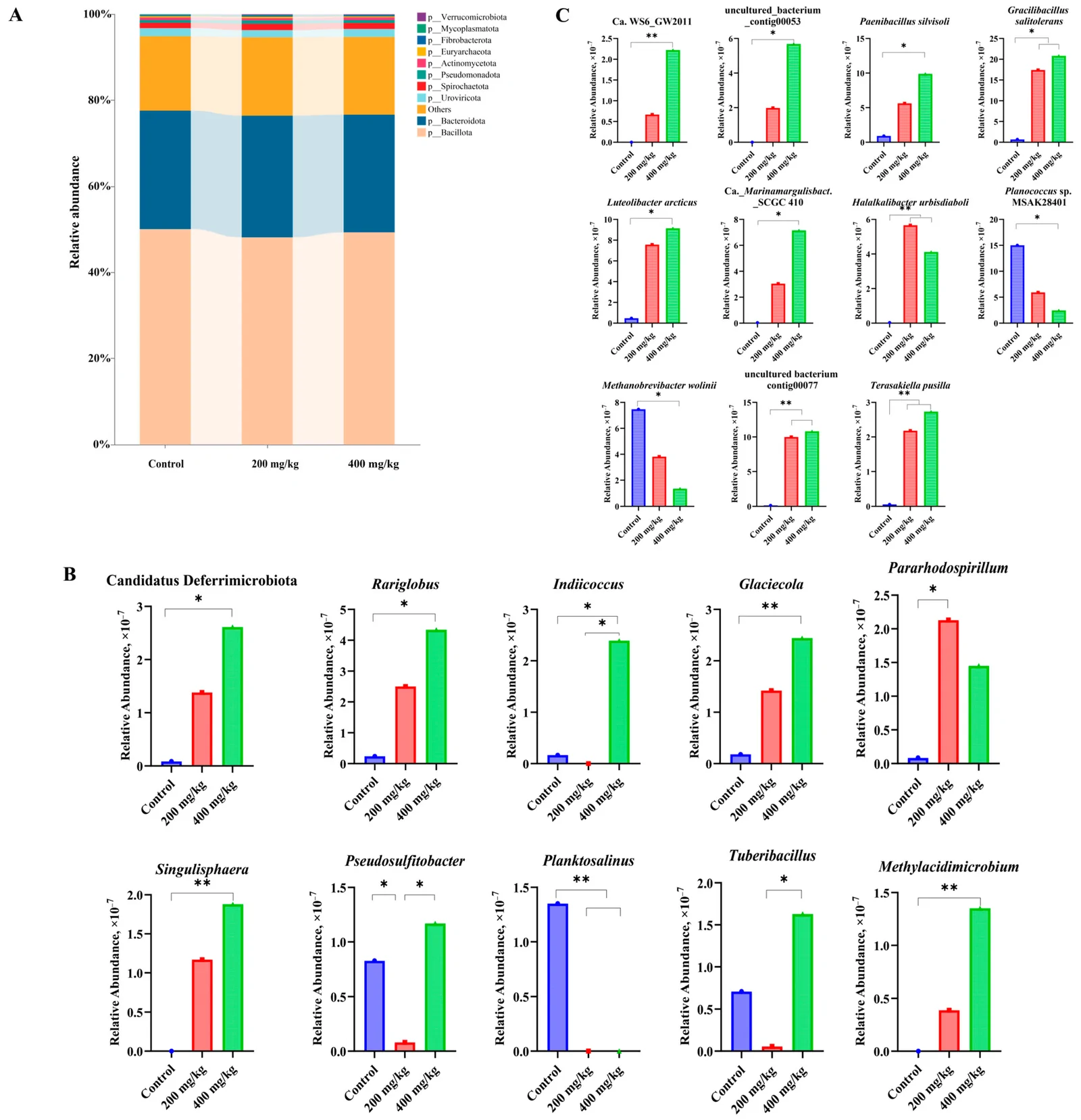
Effects of crude L-β-galactoglucan on gut microbial composition in finishing pigs (*n* = 8). (**A**) Stacked bar plot showing the relative abundances of the top 10 microbial phyla in colonic digesta across dietary treatments (control, 200 mg/kg, and 400 mg/kg groups); (**B**) bar plots illustrating differential taxa identified by MetagenomeSeq (*Q* < 0.05). Relative abundances of selected differential taxa at the phylum (Candidatus Deferrimicrobiota) and genus (*Rariglobus*, *Indiicoccus*, *Glaciecola*, *Pararhodospirillum*, *Singulisphaera*, Pseudosulfitobacter, Planktosalinus, *Tuberibacillus*, *Methylacidimicrobium*). (**C**) Bar plots of differential species including GW2011_GWF2_39_15, uncultured bacterium contig00053, *Paenibacillus silvisoli*, *Gracilibacillus salitolerans*, *Luteolibacter arcticus*, Candidatus *Marinamargulisbacteria* bacterium SCGC AG−410−N11, *Halalkalibacter urbisdiaboli*, *Planococcus* sp. MSAK28401, *Methanobrevibacter wolinii*, uncultured bacterium contig00077, and Terasakiella pusilla. Bars represent mean values with corresponding variation. Asterisks indicate significant differences (* *Q* < 0.05; ** *Q* < 0.01).

Metagenomic analysis revealed that the control, the 200 mg/kg, and the 400 mg/kg groups exhibited similar relative compositions at the phylum level, with the *Bacillota* and *Bacteroidetes* being the dominant phyla (Figure 3A). The relative abundances of *Bacillota* were 50.01%, 48.17%, and 49.32%, respectively, and those of the *Bacteroidetes* were 27.52%, 28.26%, and 27.35%, indicating that crude L-β-galactoglucan did not significantly alter the dominant phyla. At the genus level, compared with the control group, the 400 mg/kg group significantly increased the relative abundance of several rare genera, including *Rariglobus*, *Indiicoccus*, *Glaciecola*, *Singulisphaera*, *Tuberibacillus* and *Methylacidimicrobium*, while significantly reducing the relative abundance of *Planktosalinus* (Figure 3B). Additionally, the 200 mg/kg group significantly increased *Pararhodospirillum* and decreased *Pseudosulfitobacter* and *Planktosalinus* (Figure 3B). Different supplemental dosages exerted distinct regulatory effects on low-abundance intestinal genera. At the species level, the 400 mg/kg group significantly enriched the following microorganisms: candidate division WS6 bacterium GW2011_GWF2_39_15, uncultured bacterium contig00053, *Paenibacillus silvisoli*, *Gracilibacillus salitolerans*, and *Luteolibacter arcticus*; Candidatus Marinamargulisbacteria bacterium SCGC AG-410-N11; and *Halalkalibacter urbisdiaboli*, while significantly reducing the relative abundance of *Planococcus* sp. MSAK28401 and *Methanobrevibacter wolinii*. Moreover, the 200 mg/kg group enriched certain functionally related bacterial species, including uncultured bacterium contig00077, *Halalkalibacter urbisdiaboli*, *Gracilibacillus salitolerans* and *Terasakiella pusilla* (Figure 3C). This further suggests that this polysaccharide may alter the profiles of low-abundance intestinal microbes in a dose-dependent manner.

#### 3.4.3. The Effect of Crude L-β-Galactoglucan on Functional Annotation of Gut Microbiota

**Figure 4:**
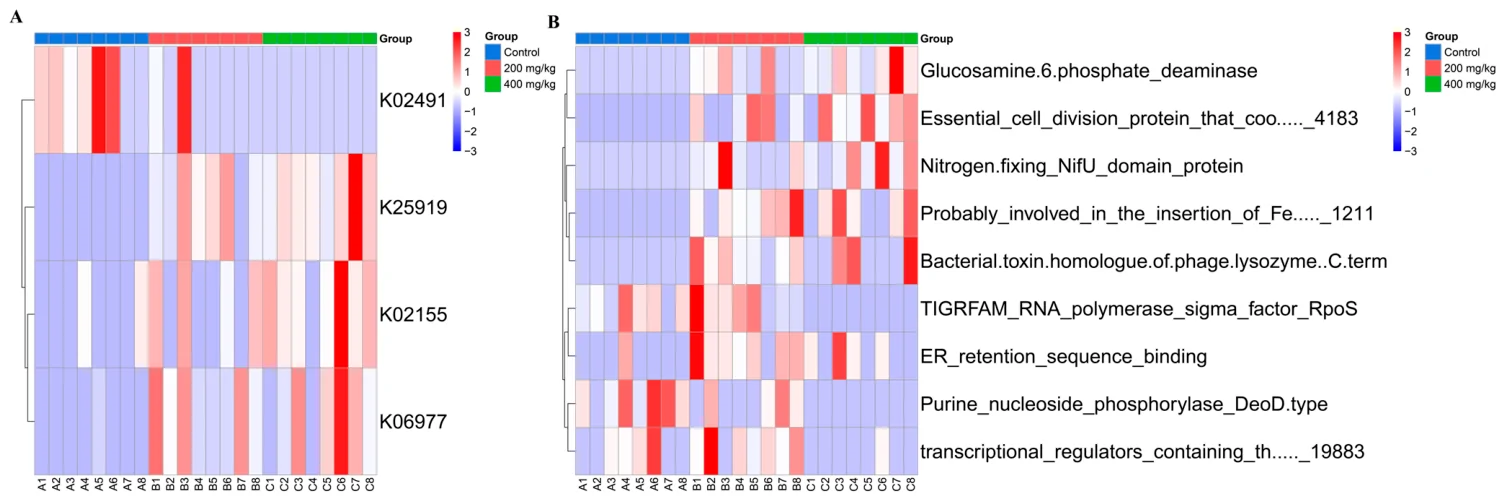
Effects of crude L-β-galactoglucan on functional profiles of gut microbiota in finishing pigs based on KEGG and eggNOG annotations (*n* = 8). (**A**) Heatmap of KEGG Orthology (KO) terms. Rows represent selected KO identifiers corresponding to functional pathways or gene functions, and columns represent individual samples from three dietary groups: control, 200 mg/kg, and 400 mg/kg groups. Color intensity indicates the normalized relative abundance of each KO term, ranging from −3 (blue, low abundance) to 3 (red, high abundance). (**B**) Heatmap of eggNOG functional annotations. The three labels with truncated names correspond to: (1) transcriptional regulators containing th(…)_19883: transcriptional regulators containing the CopG Arc MetJ DNA-binding domain and a metal-binding domain; (2) Probably involved in the insertion of Fe(…)_1211: Probably involved in the insertion of Fe-S clusters into apoproteins in vivo including IspG and or IspH. Essential for growth under aerobic conditions and for anaerobic respiration but not for fermentation. In vitro it binds Fe-S clusters and transfers them to apo-IspG, which is involved in quinone biosynthesis among many other cell components. Experiments indicate that it is probably also involved in the insertion of other Fe-S clusters than IspG IspH; (3) Essential cell division protein that coo(…)_4183: Essential cell division protein that coordinates cell division and chromosome segregation. The N-terminus is involved in assembly of the cell-division machinery. The C-terminus functions as a DNA motor that moves dsDNA in an ATP-dependent manner towards the difSL recombination site, which is located within the replication terminus region. Rows represent annotated functional gene descriptions and columns correspond to the same samples as in (**A**). Color intensity represents normalized relative abundance using the same scale as in (**A**).

Functional annotation revealed that crude L-β-galactoglucan, particularly at the 400 mg/kg dose, was associated with increased relative abundance of genes involved in several key metabolic functions, including Solabiose phosphorylase (K25919), V-type H^+^ -transporting ATPase 16 kDa proteolipid subunit (K02155), and two-component system (Figure 4A). This finding indicated that the 400 mg/kg treatment corresponded to increased predicted gene abundance related to carbohydrate and energy metabolism of intestinal microorganisms. In contrast, the abundance of sporulation sensor kinase A (K02491) decreased in the 400 mg/kg group (Figure 4A), suggesting that high-dose crude L-β-galactoglucan supplementation was accompanied by lower predicted abundance of genes involved in microbial stress response and sporulation.

#### 3.4.4. Effects of Crude L-β-Galactoglucan on the Annotation of eggNOG Functional Proteins

At the eggNOG annotation level, crude L-β-galactoglucan also exhibited dose-dependent changes in protein expression. At 400 mg/kg, crude L-β-galactoglucan significantly increased the relative abundance of essential cell division protein-4183, NifU protein, and Glucosamine-6-phosphate deaminase, while significantly reducing the relative abundance of RpoS and Purine nucleoside phosphorylase (DeoD-type). The 200 mg/kg group significantly enriched the relative abundance of Glucosamine-6-phosphate deaminase, Bacterial toxin homolog of phage lysozyme, and ER retention sequence binding (Figure 4B).

### 3.5. Intestinal Short-Chain Fatty Acid Analysis

GC-FID results showed that acetate and propionate were the main SCFA components in colonic digesta, followed by butyrate. No significant intergroup differences were observed for acetate, propionate, and butyrate. Compared with the control group, the 400 mg/kg group significantly increased the levels of isobutyric acid and isovaleric acid (*p* < 0.001, Table 6). As typical metabolites from microbial protein and amino acid fermentation, the altered branched-chain fatty acid concentrations implied that crude L-β-galactoglucan could modulate intestinal microbial nitrogen metabolism, which was further supported by subsequent functional metagenomic analysis.

### 3.6. Association Analysis Between Intestinal Short-Chain Fatty Acids and Microbiota

**Figure 5:**
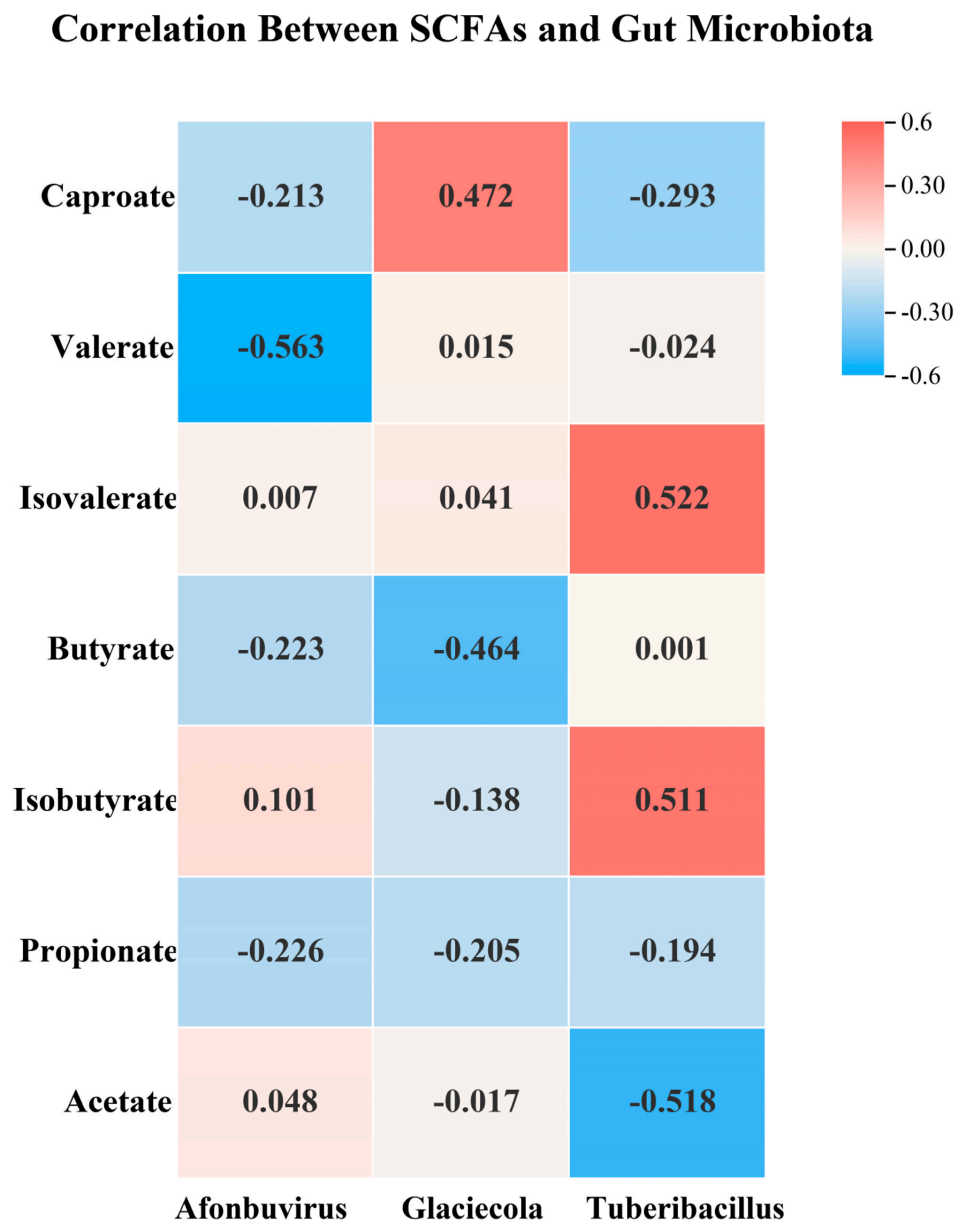
Correlation heatmap between short−chain fatty acids (SCFAs) and gut microbiota. The correlation analysis between microbial taxa and short-chain fatty acids was performed using all 18 samples from the three treatment groups. Rows represent individual SCFAs (caproate, valerate, isovalerate, butyrate, isobutyrate, propionate, and acetate), and columns represent selected microbial taxa (Afonbuvirus, Glaciecola, and Tuberibacillus). Color intensity indicates the Spearman correlation coefficient, ranging from −0.6 (blue, strong negative correlation) to 0.6 (red, strong positive correlation). Values within the cells are the correlation coefficients.

To further elucidate the relationship between crude L-β-galactoglucan-mediated gut microbiota changes and SCFA metabolism, Spearman correlation and treatment-controlled partial-correlation analyses were performed on differential microbiota and SCFA concentrations. Several microbe–SCFA pairs exhibited nominal significance at the raw *p*-value level, including associations of Tuberibacillus, Glaciecola, and Afonbuvirus with specific SCFAs (Figure 5, Supplementary Table S3). However, after FDR correction for multiple comparisons, none of these associations remained statistically significant. Partial correlation analysis, in which inter-treatment variation was accounted for, further confirmed that no robust correlations existed between microbial taxa and SCFA concentrations within each treatment group.

## 4. Discussion

In recent years, growing attention has been paid to host gut microecological balance and safe natural bioactive molecules. Gut homeostasis regulation has become a core research hotspot for natural biological modulators. Prebiotic polysaccharides, due to their unique structural stability and digestive tolerance, can reach the colon intact and be utilized by the microbiota, thereby playing a significant role in regulating the gut environment and improving host nutrient metabolism. Previous studies have shown that β-glucan, as a typical dietary fiber, is largely indigestible in the small intestine and is primarily fermented by the colonic microbiota to produce short-chain fatty acids, thereby improving intestinal barrier function and immune status [8,14].

In this study, HPSEC-RI analysis showed that L-β-galactoglucan was resistant to digestive enzymes under in vitro conditions. Although polysaccharides generally exhibit digestive resistance, their physicochemical properties (e.g., molecular weight, polymerization degree, glycosidic bond type, and branched structure) have been reported to influence fermentation rates, microbial selectivity, and metabolic product composition [9,15]. These differences may lead to distinct effects on the gut microbiota. Therefore, crude L-β-galactoglucans may regulate microbial community composition and metabolic activity through their structural characteristics, which could contribute to improvements in intestinal health and growth performance.

Previous studies have shown that β-glucans derived from microorganisms (including yeast, bacteria, and algae) can reduce pathogenic bacteria, enhance immune responses, and improve intestinal health and growth performance in weaned piglets [7,16]. However, compared with yeast- and algae-derived β-glucans, studies on bacterial-derived β-glucans remain limited. β-glucan derived from *Agrobacterium* sp. ZX09 has been reported to improve growth performance and intestinal function in weaned piglets and finishing pigs [17,18,19]. In the present study, a long-term feed trial demonstrated that supplementation with 400 mg/kg improved growth performance, as indicated by increased daily gain and final body weight, along with a lower F/G ratio compared to diets without supplementation. Additionally, apparent digestibility of DM, CP, EE, and GE was increased in the groups supplemented with 200 mg/kg and 400 mg/kg, suggesting that the observed growth-promoting effects were associated with enhanced nutrient utilization. These findings are consistent with previous studies showing that β-glucans can improve the growth performance and nutrient digestibility in pigs [20]. Consistent with the significant Treatment × Day interaction for body weight observed in linear mixed model analysis, supplementation with crude L-β-galactoglucan at 200 mg/kg and 400 mg/kg modulated the growth performance of pigs via distinct pathways, and such responses exhibited obvious stage-dependent effects. During the late finishing period (d 154–196), the ADG of the 200 mg/kg group was numerically higher than that of the 400 mg/kg group, although no significant difference was detected between treatments. Notably, the ADG of pigs receiving 200 mg/kg increased markedly from the mid-finishing stage to the late finishing stage (from 589.01 to 781.38 g/d). The 400 mg/kg supplementation level exerted growth-promoting effects through improving feed efficiency and nutrient digestibility. In the nursery and early-to-middle growing-finishing stages, ADFI showed minor differences between the two treatments. Nutrient utilization efficiency served as the core limiting factor for growth, and superior performance was observed in the 400 mg/kg group during these periods. In the late finishing stage, divergent growth trends emerged between doses, resulting in numerically greater ADG in the 200 mg/kg group. Collectively, these findings suggest that low and high supplemental doses of crude L-β-galactoglucan differ in their regulatory mechanisms for growth, and there is no single optimal dose applicable to the whole growth cycle of pigs.

Metagenomic analysis revealed that crude L-β-galactoglucan did not significantly alter the dominant bacterial phyla, such as Bacillota and Bacteroidetes, across different supplementation levels, suggesting relative stability in the overall community structure. However, differential effects were observed at the level of low-abundance taxa. MetaGenomeSeq analysis indicated that the 400 mg/kg supplementation group increased the abundance of Candidatus Deferri*microbiota* and several rare genera (e.g., *Singulisphaera*, *Glaciecola*, *Methylacidimicrobium*), while reducing the relative abundance of *Planktosalinus*. At the species level, the 400 mg/kg treatment group enriched *Paenibacillus silvisoli*, *Gracilibacillus salitolerans*, and *Luteolibacter arcticus*, and significantly inhibited the methanogenic bacterium *Methanobrevibacter wolinii*. Additionally, the 200 mg/kg treatment group exhibited enrichment of several functionally related taxa, including *Halalkalibacter urbisdiaboli* and *Terasakiella pusilla*. These results suggest that crude L-β-galactoglucan may modulate the intestinal microbiota primarily through selective regulation of low-abundance taxa rather than directly dominant bacterial groups. Although the functional roles of these taxa in the pig intestine remain largely unclear, previous studies have reported that some of these genera possess capacities related to polysaccharide degradation and fermentation metabolism. For example, *Singulisphaera* has been reported to degrade complex polysaccharides [21,22], and a polysaccharide utilization locus associated with Ulvan degradation has been identified in *Glaciecola*, indicating its potential for utilizing sulfated polysaccharides [23]. In addition, *Methylacidimicrobium* has been characterized as a methanotrophic bacterium with methane and hydrogen metabolic pathways [24]. Taken together, the structural characteristics of L-β-galactoglucan may provide selective ecological advantages for specific microbial taxa, thereby indirectly influencing intestinal metabolic processes and host nutrient utilization. Future studies are needed to verify these functional roles through metabolic prediction, functional annotation, or microbial isolation.

Functional annotation analysis indicated that the 400 mg/kg supplementation group increased the predicted relative abundance of selected functional genes. Specifically, genes related to Solabiose phosphorylase, V-type H-transporting ATPase, and the two-component system were enriched. V-type H-transporting ATPase is an important proton pump system involved in maintaining intracellular pH and regulating proton transmembrane transport. Although most studies have focused on eukaryotic systems, its role in microbial pH homeostasis has also been recognized [25,26]. Two-component systems, such as KdpD/KdpE, have been reported to sense environmental signals including potassium concentration, osmotic pressure, and cellular energy status, thereby regulating ion transport and stress adaptation [27,28]. At the eggNOG functional annotation level, the 400 mg/kg group increased the abundance of several metabolism-related functions, including essential cell division protein, NifU protein, and glucosamine-6-phosphate deaminase. These changes may indicate enhanced microbial growth potential, Fe–S cluster biosynthesis, and amino sugar metabolism [29,30]. Additionally, decreased abundance of RpoS and Purine nucleoside phosphorylase (DeoD-type) may reflect reduced stress response and a more stable metabolic homeostasis and state of the microbial community [31]. In contrast, although the 200 mg/kg group also showed enrichment of certain metabolic functions, the magnitude of these changes was lower than that observed in the 400 mg/kg group. This suggests that higher supplementation levels have a greater impact on microbial functional modulation.

GC-FID analysis showed that acetate and propionate were the primary short-chain fatty acids in colonic digesta, followed by butyrate. Supplementation with 400 mg/kg increased the concentration of isobutyrate and isovalerate. Previous studies have shown that acetate serves as a substrate for multiple metabolic pathways, propionate can be utilized by the liver for gluconeogenesis, and butyrate acts as a primary energy source for colonic epithelial cells, supporting epithelial cell integrity and barrier function [32]. In addition, inulin-type fructans have been reported to enhance short-chain fatty acid production, particularly butyrate [33]. Isobutyrate and isovalerate are branched-chain fatty acids primarily derived from microbial fermentation of amino acids and are typically present at very low concentrations in the blood [34]. Their production is influenced by dietary composition, particularly protein intake, and by metabolic characteristics of the gut microbiota [35]. The increased levels of these metabolites in the present study may indicate that crude L-β-galactoglucans influenced not only the regulation of carbohydrate fermentation but also amino acid metabolism within the microbial community. These findings suggest that crude L-β-galactoglucans may modulate the metabolic profile of the gut microbiota through mechanisms that differ from those of conventional prebiotics such as β-glucans, inulin, and fructooligosaccharides.

Analysis of the association between short-chain fatty acids and the microbiota showed that Tuberibacillus exhibited nominal positive associations with BCFAs at the raw *p*-value level, although these associations did not remain significant after Benjamini–Hochberg FDR correction. This observation hints that this genus might be potentially involved in protein degradation-related metabolism. Previous studies have demonstrated that some proteolytic microbes can produce isobutyrate and isovalerate via amino acid catabolism. It is speculated that crude L-β-galactoglucan might favor the proliferation or metabolic activity of such taxa, though this inference requires further experimental validation. In addition, Glaciecola showed nominal raw-*p*-level correlation with caproate, which may tentatively indicate altered functional niches of gut microbiota. Collectively, these correlative observations imply that crude L-β-galactoglucan could drive potential metabolic rearrangement toward multi-substrate and multi-pathway patterns in the intestinal microbiota, which remains to be verified by functional assays.

## 5. Conclusions

In summary, this study was designed to investigate the in vitro enzyme resistance of *Agrobacterium* sp. FN01-derived L-β-galactoglucan, as well as the effects of the corresponding crude product (dominated by L-β-galactoglucan) on growth performance, nutrient utilization, and intestinal microbiota in pigs. Collectively, the present study confirmed that L-β-galactoglucan was resistant to hydrolysis by α-amylase and glucoamylase under in vitro conditions. In pig trials, this crude L-β-galactoglucans improved growth performance, enhanced apparent nutrient digestibility, reshaped gut microbiota, and altered the concentrations of branched-chain fatty acids (BCFAs) in colon contents, including isobutyric acid and isovaleric acid. These findings provided novel theoretical evidence for its fundamental research and potential development as a natural bioactive compound. This work highlighted the intestinal regulatory potential of bacterially derived galactoglucan and offered new insights for exploring natural microecological modulators.

## Figures and Tables

**Table 1:** Dietary composition and nutritional levels during the nursery and finishing period.

Ingredient	Nursery Period	Ingredient	Finishing Period
Corn (first grade)	35.5	Corn	55
Broken rice Grade A 7%	10	Flour	7.5
Flour Grade 1 12.5% + ash 1.1%	20	Bran	5
Soybean meal 43%	12.4	Wheat bran	1.7
Fermented soybean meal 50%	4	Soybean meal	18.8
Soybean enzyme-hydrolyzed protein 52%	5	Full-fat rice bran	5
Expanded flaxseed	1	Rice bran meal	2
Soybean oil	2.1	Compound premix ^2^	5
Compound premix ^1^	10	Total	100
Calculated nutrient level		Calculated nutrient level	
Digestible energy, MJ/kg	14.31	Digestible energy, MJ/kg	13.18
Crude protein, %	17.8	Crude protein, %	15.88
Calcium, %	0.6	Calcium, %	0.65
Total phosphorus, %	0.51	Total phosphorus, %	0.57
Available phosphorus, %	0.26	Available phosphorus, %	0.22
Lysine, %	1.2	Lysine, %	1.05
Methionine, %	0.36	Methionine, %	0.31
Threonine, %	0.85	Threonine, %	0.85
		Analyzed nutrient level (air-dry basis)	
		Dry matter (DM), %	84.97
		Crude protein (CP), %	16.23
		Ether extract (EE), %	1.18
		Gross energy (GE), MJ/kg	15.70

^1^ The premix provided the following per kilogram of the diet: vitamin A, 4500 IU; vitamin D_3_, 2250 IU; vitamin E, 25 IU; vitamin K, 3 mg; vitamin B_1_, 1.8 mg; vitamin B_6_, 15 mg; vitamin B_12_, 25 ug; riboflavin, 8 mg; folic acid, 0.9 mg; biotin, 0.5 mg; niacin, 24 mg; pantothenic acid, 20 mg; Zn (as zinc oxide), 100 mg; Fe (as ferrous sulfate), 150 mg; Cu (as copper sulfate), 10 mg; Mn, 5 mg; I (as potassium iodide), 0.6 mg; Se (as sodium selenite), 0.5 mg; Co (as cobalt chloride), 0.4 mg. ^2^ The premix provided the following per kilogram of the diet: vitamin A, 3450 IU; vitamin D_3_, 1250 IU; vitamin E, 25 IU; vitamin K, 2 mg; vitamin B_1_, 1.8 mg; vitamin B_6_, 5 mg; vitamin B_12_, 12 ug; riboflavin, 3 mg; folic acid, 0.5 mg; biotin, 0.5 mg; niacin, 15 mg; pantothenic acid, 15 mg; Zn (as zinc oxide), 80 mg; Fe (as ferrous sulfate), 120 mg; Cu (as copper sulfate), 10 mg; Mn, 4 mg; I (as potassium iodide), 0.5 mg; Se (as sodium selenite), 0.5 mg; Co (as cobalt chloride), 0.2 mg. Compound premix ingredients: Inorganic trace-mineral premix, vitamin premix, carrier, sweetener, and antioxidant. The dietary supply levels of vitamins and trace minerals are fully provided in footnotes ^1^ and ^2^. Analyzed nutrients were only determined for finishing-period diets corresponding to colonic digestibility assay. Nutrient data for growing-phase diets are calculated values. Only crude protein, ether extract and gross energy were measured. Mean values are shown in the Table cells. The raw analytical data in the order of control (0 mg/kg), 200 mg/kg and 400 mg/kg are as follows: crude protein (16.28%, 16.31%, 16.10%), ether extract (1.18%, 1.02%, 1.33%), and gross energy (15.70 MJ/kg, 15.67 MJ/kg, 15.73 MJ/kg).

**Table 2:** Molecular weight distribution and digestibility of L-β-galactoglucan before and after in vitro enzymatic digestion.

Sample	Treatment	Polymer Peak, %	Salt and Sugar Peak, %	Mn (kDa)	Mw (kDa)	PDI
1	Undigested	87	0	31	425	14
	Digested	85	3	35	408	12
2	Undigested	88	0	31	424	14
	Digested	86	2	32	419	13
3	Undigested	88	0	30	520	18
	Digested	85	2	29	506	17

Samples 1, 2, and 3 in Table 2 represent independent production batches. Polymer peak, %: percentage of the polysaccharide fraction detected; Salt and sugar peak, %: percentage of low-molecular-weight impurities (salts, monosaccharides); Mn (kDa): number-average molecular weight; Mw (kDa): weight-average molecular weight; PDI: polydispersity index (Mw/Mn), indicating the breadth of molecular weight distribution. The system was calibrated with pullulan and disaccharide standards of known molecular mass, and the weight-average (Mw) and number-average (Mn) molecular masses were calculated relative to this calibration.

**Table 3:** Effects of crude L-β-galactoglucan on growth of pigs during the nursery-fattening period (*n* = 16).

Item	Control	200 mg/kg	400 mg/kg	SEM	*p*-Value
BW (kg)					
Initial BW	6.89	6.89	6.89	0.192	1.000
D37	8.21	8.19	8.14	0.184	0.988
D66	19.19	19.34	19.57	0.283	0.857
D97	34.34 ^y^	35.65 ^xy^	36.08 ^x^	0.343	0.095
D153	68.48 ^b^	69.23 ^b^	77.35 ^a^	0.61	<0.0001
D197	97.31 ^c^	102.83 ^b^	108.92 ^a^	1.061	<0.0001
Main effect					
Treatment					<0.0001
Time					<0.0001
Treatment × Time					<0.0001
ADG (g/d)					
D37–65	378.41	384.45	394.23	4.211	0.445
D66–96	488.79 ^b^	526.22 ^a^	532.58 ^a^	7.251	0.025
D97–153	599.06 ^b^	589.01 ^b^	723.95 ^a^	11.391	<0.0001
D154–196	670.37 ^b^	781.38 ^a^	734.31 ^a^	13.233	0.001
D66–196	596.37 ^c^	637.29 ^b^	682.06 ^a^	7.624	<0.0001
ADFI (kg/d)					
D37–65	0.60 ^ab^	0.64 ^a^	0.58 ^b^	0.007	0.003
D66–96	1.16	1.20	1.19	0.012	0.331
D97–153	2.02 ^b^	2.03 ^b^	2.28 ^a^	0.028	<0.0001
D154–196	2.99	3.08	2.97	0.031	0.484
D66–196	2.13	2.21	2.25	0.022	0.103
F/G					
D37–65	1.59 ^b^	1.66 ^a^	1.47 ^c^	0.016	<0.0001
D66–96	2.38 ^x^	2.28 ^xy^	2.25 ^y^	0.024	0.052
D97–153	3.39 ^a^	3.45 ^a^	3.16 ^b^	0.035	0.001
D154–196	4.49 ^a^	3.99 ^b^	4.06 ^b^	0.071	0.005
D66–196	3.58 ^a^	3.47 ^b^	3.29 ^c^	0.024	<0.0001

BW = body weight; ADG = average daily gain; ADFI = average daily feed intake; F/G = feed-to-gain ratio. Different superscript letters (a, b, c) within the same row indicate significant differences between treatment groups. Different superscript letters (x, y) within the same row indicate a tendency for difference. *p* ≤ 0.05, the difference is significant; 0.05 < *p* ≤ 0.10, there is a trend difference; *p* > 0.05, the difference is not significant.

**Table 4:** Apparent digestibility of feed in the intestines of finishing pigs (*n* = 10).

	Control	200 mg/kg	400 mg/kg	SEM	*p*-Value
Dry matter, %	80.37 ^b^	84.08 ^a^	83.26 ^a^	0.392	0.001
Crude fat, %	56.39 ^b^	61.14 ^b^	68.19 ^a^	1.428	0.001
Crude protein, %	81.57 ^b^	85.77 ^a^	85.24 ^a^	0.408	0.001
Gross energy, %	82.83 ^b^	85.81 ^a^	85.37 ^a^	0.282	0.001

Different superscript letters (a, b) within the same row indicate significant differences between treatment groups. *p* ≤ 0.05, the difference is significant; 0.05 < *p* ≤ 0.10, there is a trend difference; *p* > 0.05, the difference is not significant.

**Table 5:** Analysis of intestinal microbial diversity in finishing pigs (*n* = 8).

	Control	200 mg/kg	400 mg/kg	SEM	*p*-Value
Phylum					
ACE	158.46 ^y^	166.50 ^x^	160.51 ^xy^	1.383	0.057
chao1	158.26 ^b^	167.06 ^a^	160.07 ^b^	1.444	0.030
Shannon	0.99	1.02	1.00	0.012	0.504
Simpson	0.53	0.54	0.53	0.004	0.554
Observed species	157.00	164.50	159.63	1.372	0.109
Genus					
ACE	2705.03	2914.95	2772.31	38.811	0.108
chao1	2713.45 ^y^	2936.19 ^x^	2774.63 ^xy^	40.354	0.074
Shannon	4.02	4.05	4.10	0.022	0.348
Simpson	0.95	0.95	0.96	0.001	0.386
Observed species	2650.13	2853.50	2733.50	38.456	0.155
Species					
ACE	12,014.05	12,905.79	12,145.74	178.916	0.130
chao1	12,055.51	13,016.04	12,148.13	190.847	0.101
Shannon	5.87	5.94	6.04	0.033	0.119
Simpson	0.987 ^y^	0.989 ^xy^	0.991 ^x^	0.006	0.096
Observed species	11,635.25	12,476.38	11,876.63	168.039	0.205

ACE: Estimates the potential richness of taxa within the community. Chao1: Estimates the total number of taxa in samples. Shannon index reflects community diversity by integrating the richness and relative abundance of taxa. Simpson index characterizes community diversity and the evenness of taxon distribution. Observed species represents the number of detected taxa. Different superscript letters (a, b) within the same row indicate significant differences between treatment groups. Different superscript letters (x, y) within the same row indicate a tendency for difference. *p* ≤ 0.05, the difference is significant; 0.05 < *p* ≤ 0.10, there is a trend difference; *p* > 0.05, the difference is not significant.

**Table 6:** Effects of L-crude β-galactoglucan on the content of short-chain fatty acids in the colonic digesta of finishing pigs (*n* = 6).

	Control	200 mg/kg	400 mg/kg	SEM	*p*-Value
Acetic acid, μmol/g	6.09	7.00	5.64	0.367	0.109
Propionic acid, μmol/g	6.34	6.86	5.76	0.309	0.133
Isobutyric acid, μmol/g	0.37 ^b^	0.28 ^c^	0.44 ^a^	0.025	<0.001
Butyrate, μmol/g	3.94	3.79	3.58	0.273	0.519
Isovaleric acid, μmol/g	0.54 ^b^	0.38 ^c^	0.73 ^a^	0.052	<0.001
Valeric acid, μmol/g	0.54	0.64	0.53	0.036	0.240

Different superscript letters (a, b, c) within the same row indicate significant differences between treatment groups. *p* ≤ 0.05, the difference is significant; 0.05 < *p* ≤ 0.10, there is a trend difference; *p* > 0.05, the difference is not significant.

## Data Availability

The data presented in this study are available in the article. The raw metagenomic sequencing reads generated in this study have been deposited in the NCBI Sequence Read Archive (SRA) under BioProject accession number PRJNA1506119.

## Supplementary Materials

The following supporting information can be downloaded at: https://www.mdpi.com/article/10.3390/ani16172752/s1; Table S1: Effects of crude L-β-galactoglucan on body weight in weaned piglets; Table S2: Number of pigs per pen across different experimental time points, including mortalities and discontinued pigs; Table S3: Outputs of Spearman’s rank order correlation and partial correlation analyses between gut microbial taxa and short chain fatty acid (SCFA) concentrations; Table S4: Statistics of raw and clean PE150 sequencing data after fastp quality control; Table S5: PERMANOVA and PERMDISP analyses based on Bray Curtis distances for bacterial community structure at the phylum, genus, and species levels; Table S6: Complete output of differential abundance analysis for bacterial phyla, genera, species, and KEGG KO and eggNOG functional gene profiles.
