# Supplementary material for: Supplementation with Agrobacterium sp. FN01-Derived Crude Product Dominated by L-β-Galactoglucan Enhances Growth Performance and Nutrient Utilization and Modulates Intestinal Microbiota in Pigs
Source: Animals (Basel). 2026 Sep 2;16(17):2752. doi: 10.3390/ani16172752 (PMC13565666; doi:10.3390/ani16172752)
Supplement: Supplementary file 1 [file animals-16-02752-s001.zip › animals-4444472-supplementary.pdf]

Supplementary Table S1. Effects of crude L-β-galactoglucan on body weight in weaned piglets.

	Control	200 mg/kg	400 mg/kg	600 mg/kg	SEM	P-value
BW (kg)						
BW0	7.47	7.45	7.48	7.48	0.060	0.965
BW28	17.87b	17.96b	18.35a	17.54c	0.097	< 0.001
BW42	23.22c	23.64b	24.21a	22.63c	0.203	< 0.001
ADFI (kg/d)						
D0-28	0.73	0.76	0.78	0.74	0.02	0.230
D28-42	0.93	1.00	1.01	0.99	0.03	0.052
D0-42	0.82	0.86	0.87	0.84	0.03	0.293
ADG (g/d)						
D0-28	371.38	378.50	392.30	359.38	10.611	0.757
D28-42	382.60	408.08	419.29	401.29	16.693	0.804
D0-42	375.12	388.36	401.29	365.65	11.495	0.731

BW = body weight; ADG = average daily gain; ADFI = average daily feed intake.

Different superscript letters (a, b, c) within the same row indicate significant differences between treatment groups. *P* value ≤ 0.05, the difference is significant; 0.05 < *P* value ≤ 0.10, there is a trend difference; *P* value > 0.05, the difference is not significant.

Supplementary Table S2. Number of pigs per pen across different experimental time points, including mortalities and discontinued pigs.

	Treatment	D37	Mortality (D37-66)	Discontinued (D66)	D66	Mortality (D66-196)	D196
1	Control	13			13		13
2	Control	13			13		13
3	Control	13			13		13
4	Control	13			13		13
5	Control	13			13		13
6	Control	13			13		13
7	Control	13			13	2	11
8	Control	13			13		13
9	Control	13		1	12	1	11
10	Control	13			13		13
11	Control	13			13		13
12	Control	13		2	11	1	10
13	Control	13		3	10	1	9
14	Control	13		2	11		11
15	Control	13			13		13
16	Control	13		1	12	1	11
1	200 mg/kg	13			13		13
2	200 mg/kg	13		1	12		12
3	200 mg/kg	13			13		13
4	200 mg/kg	13	1		12		12
5	200 mg/kg	13			13		13
6	200 mg/kg	13	1		12		12
7	200 mg/kg	13		1	12	1	11
8	200 mg/kg	13			13		13
9	200 mg/kg	13			13		13
10	200 mg/kg	13			13		13
11	200 mg/kg	13			13		13
12	200 mg/kg	13		1	12		12
13	200 mg/kg	13		1	12		12
14	200 mg/kg	13			13		13
15	200 mg/kg	13	1		12		12
16	200 mg/kg	13			13	1	12
1	400 mg/kg	13			13		13
2	400 mg/kg	13			13		13
3	400 mg/kg	13		1	12	1	11
4	400 mg/kg	13			13		13
5	400 mg/kg	13			13		13
6	400 mg/kg	13		1	12		12
7	400 mg/kg	13			13		13
8	400 mg/kg	13		1	12		12

9	400 mg/kg	13		1	12		12
10	400 mg/kg	13		1	12		12
11	400 mg/kg	13	1		12		12
12	400 mg/kg	13		1	12	1	11
13	400 mg/kg	13		4	9		9
14	400 mg/kg	13		1	12		12
15	400 mg/kg	13	1	1	11	1	10
16	400 mg/kg	13	1		12		12

D37: number of pigs per pen at the start of formal experiment (day 37); D66: number of pigs entering the finishing-phase measurement at day 66; D196: number of surviving pigs at the end of experiment (day 196); Discontinued (D66): pigs with body weight below 15 kg at D66. These animals were removed from the pen and included for D37–66 performance calculation, but excluded from finishing-phase indices; No entire pen was excluded for statistical analysis.

Supplementary Table S3. Outputs of Spearman's rank-order correlation and partial correlation analyses between gut microbial taxa and short-chain fatty acid (SCFA) concentrations. ρ = Spearman correlation coefficient. P value ≤ 0.05, the difference is significant, *P* value > 0.05, the difference is not significant.

Taxon	SCFA	ρ	*P*-value	*Q*-value	ρ _partial	*P*_partial	*Q*_partial
Acetate	Candidatus_Deferrimicrobiota	-0.237	0.344	0.997	-0.267	0.317	0.812
Acetate	Naumovozyma	0.012	0.962	0.997	0.160	0.555	0.915
Acetate	Afonbuvirus	0.048	0.849	0.997	0.116	0.668	0.915
Acetate	Rariglobus	0.201	0.423	0.997	0.278	0.298	0.805
Acetate	Indiicoccus	-0.380	0.120	0.997	-0.154	0.570	0.915
Acetate	Glaciecola	-0.017	0.947	0.997	0.071	0.793	0.947
Acetate	Eldridgevirus	0.102	0.687	0.997	0.107	0.693	0.915
Acetate	Berkiella	0.093	0.714	0.997	0.109	0.688	0.915
Acetate	Pararhodospirillum	-0.061	0.811	0.997	-0.171	0.527	0.905
Acetate	Singulisphaera	-0.141	0.576	0.997	0.099	0.715	0.925
Acetate	Candidatus_Bandiella	0.364	0.137	0.997	0.191	0.479	0.884
Acetate	Cotonvirus	0.276	0.267	0.997	0.302	0.256	0.753
Acetate	Pakpunavirus	0.211	0.400	0.997	0.138	0.611	0.915
Acetate	Pseudosulfitobacter	-0.043	0.864	0.997	0.132	0.627	0.915
Acetate	Planktosalinus	0.148	0.559	0.997	0.247	0.356	0.824
Acetate	Tuberibacillus	-0.518	0.028	0.869	-0.234	0.383	0.836
Acetate	Methylacidimicrobium	-0.189	0.453	0.997	0.080	0.768	0.939
Propionate	Candidatus_Deferrimicrobiota	-0.431	0.074	0.997	-0.515	0.041	0.519
Propionate	Naumovozyma	-0.120	0.635	0.997	-0.047	0.862	0.958
Propionate	Afonbuvirus	-0.226	0.367	0.997	-0.222	0.408	0.852
Propionate	Rariglobus	-0.005	0.984	0.997	-0.025	0.927	0.984
Propionate	Indiicoccus	-0.025	0.922	0.997	0.187	0.488	0.884
Propionate	Glaciecola	-0.205	0.415	0.997	-0.255	0.341	0.818
Propionate	Eldridgevirus	-0.057	0.824	0.997	-0.138	0.611	0.915
Propionate	Berkiella	-0.093	0.714	0.997	-0.111	0.681	0.915
Propionate	Pararhodospirillum	-0.103	0.684	0.997	-0.196	0.467	0.884
Propionate	Singulisphaera	-0.222	0.375	0.997	-0.187	0.488	0.884
Propionate	Candidatus_Bandiella	0.239	0.339	0.997	0.114	0.676	0.915
Propionate	Cotonvirus	0.021	0.933	0.997	-0.019	0.945	0.992
Propionate	Pakpunavirus	-0.148	0.559	0.997	-0.243	0.365	0.836
Propionate	Pseudosulfitobacter	-0.093	0.714	0.997	0.006	0.984	0.995
Propionate	Planktosalinus	0.043	0.866	0.997	0.079	0.772	0.939
Propionate	Tuberibacillus	-0.194	0.439	0.997	0.092	0.735	0.927
Propionate	Methylacidimicrobium	-0.086	0.735	0.997	0.100	0.712	0.925
Isobutyrate	Candidatus_Deferrimicrobiota	0.127	0.616	0.997	0.133	0.623	0.915
Isobutyrate	Naumovozyma	0.100	0.692	0.997	-0.001	0.996	1.000
Isobutyrate	Afonbuvirus	0.101	0.691	0.997	0.065	0.811	0.951

Isobutyrate	Rariglobus	-0.153	0.544	0.997	-0.179	0.508	0.905
Isobutyrate	Indiicoccus	0.074	0.769	0.997	-0.210	0.434	0.872
Isobutyrate	Glaciecola	-0.138	0.586	0.997	-0.274	0.305	0.805
Isobutyrate	Eldridgevirus	0.160	0.526	0.997	0.327	0.217	0.719
Isobutyrate	Berkiella	0.195	0.439	0.997	0.238	0.374	0.836
Isobutyrate	Pararhodospirillum	0.233	0.352	0.997	0.432	0.095	0.519
Isobutyrate	Singulisphaera	-0.100	0.692	0.997	-0.483	0.058	0.519
Isobutyrate	Candidatus_Bandiella	-0.074	0.769	0.997	0.145	0.591	0.915
Isobutyrate	Cotonvirus	0.112	0.658	0.997	0.204	0.448	0.880
Isobutyrate	Pakpunavirus	-0.037	0.886	0.997	0.056	0.838	0.958
Isobutyrate	Pseudosulfitobacter	0.356	0.147	0.997	0.276	0.300	0.805
Isobutyrate	Planktosalinus	0.345	0.161	0.997	0.462	0.072	0.519
Isobutyrate	Tuberibacillus	0.511	0.030	0.869	0.298	0.263	0.765
Isobutyrate	Methylacidimicrobium	0.109	0.666	0.997	-0.137	0.613	0.915
Butyrate	Candidatus_Deferrimicrobiota	-0.356	0.147	0.997	-0.302	0.256	0.753
Butyrate	Naumovozyma	-0.427	0.077	0.997	-0.407	0.118	0.600
Butyrate	Afonbuvirus	-0.223	0.373	0.997	-0.305	0.251	0.753
Butyrate	Rariglobus	-0.281	0.260	0.997	-0.219	0.414	0.854
Butyrate	Indiicoccus	0.111	0.661	0.997	0.193	0.475	0.884
Butyrate	Glaciecola	-0.464	0.053	0.910	-0.463	0.071	0.519
Butyrate	Eldridgevirus	0.027	0.915	0.997	-0.205	0.446	0.880
Butyrate	Berkiella	-0.234	0.350	0.997	-0.347	0.188	0.691
Butyrate	Pararhodospirillum	-0.269	0.280	0.997	-0.214	0.427	0.864
Butyrate	Singulisphaera	-0.468	0.050	0.910	-0.519	0.039	0.519
Butyrate	Candidatus_Bandiella	-0.040	0.873	0.997	-0.125	0.644	0.915
Butyrate	Cotonvirus	0.003	0.991	0.997	-0.127	0.640	0.915
Butyrate	Pakpunavirus	-0.291	0.242	0.997	-0.395	0.130	0.646
Butyrate	Pseudosulfitobacter	0.212	0.397	0.997	0.201	0.456	0.884
Butyrate	Planktosalinus	0.122	0.629	0.997	0.010	0.969	0.995
Butyrate	Tuberibacillus	0.001	0.997	0.997	0.053	0.845	0.958
Butyrate	Methylacidimicrobium	-0.222	0.376	0.997	-0.109	0.688	0.915
Isovalerate	Candidatus_Deferrimicrobiota	0.290	0.243	0.997	0.260	0.331	0.818
Isovalerate	Naumovozyma	0.041	0.872	0.997	-0.124	0.648	0.915
Isovalerate	Afonbuvirus	0.007	0.979	0.997	-0.001	0.998	1.000
Isovalerate	Rariglobus	-0.003	0.990	0.997	-0.088	0.746	0.933
Isovalerate	Indiicoccus	0.082	0.745	0.997	-0.291	0.274	0.765
Isovalerate	Glaciecola	0.041	0.872	0.997	-0.175	0.516	0.905
Isovalerate	Eldridgevirus	-0.039	0.877	0.997	0.194	0.472	0.884
Isovalerate	Berkiella	0.025	0.922	0.997	0.108	0.689	0.915
Isovalerate	Pararhodospirillum	0.288	0.246	0.997	0.430	0.096	0.519
Isovalerate	Singulisphaera	0.041	0.872	0.997	-0.516	0.041	0.519
Isovalerate	Candidatus_Bandiella	-0.276	0.268	0.997	-0.032	0.907	0.971
Isovalerate	Cotonvirus	-0.052	0.836	0.997	0.105	0.698	0.918
Isovalerate	Pakpunavirus	-0.211	0.400	0.997	-0.091	0.737	0.927

Isovalerate	Pseudosulfitobacter	0.318	0.198	0.997	0.275	0.303	0.805
Isovalerate	Planktosalinus	0.170	0.500	0.997	0.360	0.171	0.685
Isovalerate	Tuberibacillus	0.522	0.026	0.869	0.256	0.339	0.818
Isovalerate	Methylacidimicrobium	0.281	0.259	0.997	-0.063	0.818	0.954
Valerate	Candidatus_Deferrimicrobiota	-0.047	0.852	0.997	-0.115	0.672	0.915
Valerate	Naumovozyma	-0.109	0.666	0.997	-0.098	0.718	0.925
Valerate	Afonbuvirus	-0.563	0.015	0.869	-0.559	0.025	0.492
Valerate	Rariglobus	-0.216	0.390	0.997	-0.358	0.173	0.685
Valerate	Indiicoccus	0.247	0.323	0.997	0.357	0.174	0.685
Valerate	Glaciecola	0.015	0.954	0.997	-0.065	0.810	0.951
Valerate	Eldridgevirus	-0.253	0.311	0.997	-0.291	0.275	0.765
Valerate	Berkiella	-0.393	0.107	0.997	-0.389	0.136	0.653
Valerate	Pararhodospirillum	0.073	0.772	0.997	-0.008	0.975	0.995
Valerate	Singulisphaera	0.040	0.875	0.997	0.007	0.978	0.995
Valerate	Candidatus_Bandiella	-0.210	0.402	0.997	-0.274	0.304	0.805
Valerate	Cotonvirus	-0.142	0.575	0.997	-0.119	0.661	0.915
Valerate	Pakpunavirus	-0.227	0.365	0.997	-0.237	0.377	0.836
Valerate	Pseudosulfitobacter	-0.022	0.930	0.997	0.051	0.851	0.958
Valerate	Planktosalinus	-0.354	0.149	0.997	-0.369	0.160	0.685
Valerate	Tuberibacillus	-0.024	0.925	0.997	0.103	0.706	0.923
Valerate	Methylacidimicrobium	0.100	0.694	0.997	0.134	0.621	0.915
Caproate	Candidatus_Deferrimicrobiota	0.548	0.019	0.869	0.460	0.073	0.519
Caproate	Naumovozyma	-0.117	0.644	0.997	-0.126	0.642	0.915
Caproate	Afonbuvirus	-0.213	0.396	0.997	-0.034	0.900	0.967
Caproate	Rariglobus	0.271	0.277	0.997	-0.030	0.912	0.972
Caproate	Indiicoccus	-0.218	0.385	0.997	-0.108	0.689	0.915
Caproate	Glaciecola	0.472	0.048	0.910	0.302	0.256	0.753
Caproate	Eldridgevirus	-0.266	0.287	0.997	0.149	0.583	0.915
Caproate	Berkiella	-0.012	0.961	0.997	0.230	0.391	0.836
Caproate	Pararhodospirillum	0.380	0.120	0.997	0.083	0.759	0.939
Caproate	Singulisphaera	0.313	0.206	0.997	0.153	0.573	0.915
Caproate	Candidatus_Bandiella	-0.124	0.623	0.997	-0.230	0.391	0.836
Caproate	Cotonvirus	-0.365	0.137	0.997	-0.217	0.420	0.856
Caproate	Pakpunavirus	0.053	0.835	0.997	0.171	0.527	0.905
Caproate	Pseudosulfitobacter	0.017	0.947	0.997	0.336	0.204	0.699
Caproate	Planktosalinus	-0.307	0.216	0.997	0.000	1.000	1.000
Caproate	Tuberibacillus	-0.293	0.237	0.997	0.007	0.980	0.995
Caproate	Methylacidimicrobium	0.386	0.114	0.997	0.434	0.093	0.519

ρ, Spearman's rank-order correlation coefficient; *P*_value, raw *P*-value of Spearman correlation; *Q*_value, FDR-adjusted *Q*-value for Spearman correlation; ϱ_partial, partial correlation coefficient; *P*_partial, raw *P*-value of partial correlation; *Q*_partial, FDR-adjusted *Q*-value for partial correlation. Correlation or partial correlation was considered statistically significant at $Q \leq 0.05$.

Supplementary Table S4. Statistics of raw and clean PE150 sequencing data after fastp quality control.

Sample	Raw_Base (G)	Clean_Base (G)	Clean_Q20 (%)	Clean_Q30 (%)	Clean_GC(%)	Effective(%)
A1	7.49	7.44	99.18	97.3	45.49	99.36
A2	6.83	6.77	99.12	97.21	46.59	99.21
A3	7.77	7.7	99.12	97.16	45.76	99.11
A4	8.26	8.12	99.1	97.14	47.7	98.32
A5	7.8	7.74	99.11	97.12	46.88	99.22
A6	7.76	7.68	99.08	97.03	47.13	98.96
A7	6.78	6.72	99.1	97.1	45.94	99.08
A8	7.97	7.92	99.1	97.22	45.89	99.35
B1	8.11	8.06	99.12	97.23	45.61	99.44
B2	7.13	7.08	99.06	97.01	45.7	99.25
B3	7.2	7.12	99.09	97.17	46.71	98.93
B4	9.65	9.58	99.11	97.25	46.23	99.32
B5	11.28	11.16	99.14	97.31	45.87	98.94
B6	9.81	9.72	99.06	97.11	46.29	99.09
B7	8.74	8.66	99.15	97.28	45.6	99.1
B8	8.74	8.64	99.12	97.26	45.8	98.85
C1	11.12	10.97	99.16	97.36	45.62	98.63
C2	7.08	7.06	99.16	97.3	45.41	99.72
C3	6.95	6.93	99.16	97.3	46.79	99.74
C4	8.36	8.28	99.11	97.24	45.86	99.13
C5	11.29	11.09	99.03	97.07	45.62	98.21
C6	6.6	6.5	99.11	97.26	45.1	98.62
C7	8.21	8.14	99.1	97.16	45.2	99.15
C8	6.87	6.76	99.03	97.16	45.06	98.42

A1-A8: Control group; B1-B8: 200 mg/kg group; C1-C8: 400 mg/kg group, A1-A6, B1-B6, C1-C6 for SCFA determination. Ten colonic contents samples were collected per treatment with global original identifiers 1-30. Eight samples per treatment were randomly selected for metagenomic sequencing and functional analysis. From these eight sequenced samples, six were further randomly selected for SCFA and digestibility assays and assigned secondary analytical labels A1-A6, B1-A6, C1-C6. The remaining two sequenced samples per treatment were labelled A7-A8, B7-B8, C7-C8 and only used for metagenomic-related analyses. All random subsampling was performed before experimental measurements without reference to any phenotypic results.

Supplementary Table S5. PERMANOVA and PERMDISP analyses based on Bray-Curtis distances for bacterial community structure at the phylum, genus, and species levels.

Taxonomic level	PERMANOVA		PERMDISP	
	F-value	*P*-value	*F*-value	*P*-value
Phylum	0.347	0.884	0.209	0.813
Genus	1.356	0.173	0.653	0.531
Species	1.399	0.099	0.954	0.386

PERMANOVA was performed to assess differences in bacterial community structure among treatment groups. PERMDISP was used to test the homogeneity of multivariate dispersion across groups. $P < 0.05$ was considered statistically significant.

Supplementary Table S6. Complete output of differential abundance analysis for bacterial phyla, genera, species, and KEGG KO and eggNOG functional gene profiles.

	**Comparison**	***P*-value**	***Q*-value**	**log_2FC**	**lfcSE**	**Prevalence**
Phylum						
Candidatus Deferrimicrobiota	Control-200mg/kg	0.016	0.980	0.884	0.366	0.500
	Control-400mg/kg	0.0002	0.039	1.551	0.418	
	200mg/kg-400mg/kg	0.585	1.000	0.378	0.693	
Genus						
Rariglobus	Control-200mg/kg	0.002	0.461	2.059	0.672	0.833
	Control-400mg/kg	0.0001	0.04	2.547	0.669	
	200mg/kg-400mg/kg	0.224	1.000	0.707	0.582	
Indiicoccus	Control-200mg/kg	0.584	1.000	-0.108	0.197	0.208
	Control-400mg/kg	0.0001	0.015	2.834	0.689	
	200mg/kg-400mg/kg	0.011	1.000	1.384	0.543	
Glaciecola	Control-200mg/kg	0.006	0.778	1.418	0.514	0.625
	Control-400mg/kg	0.001	0.003	1.635	0.343	
	200mg/kg-400mg/kg	0.734	1.000	-0.225	0.661	
Pararhodospirillum	Control-200mg/kg	0.0001	0.019	1.613	0.355	0.625
	Control-400mg/kg	0.014	0.708	1.092	0.446	
	200mg/kg-400mg/kg	1.000	1.000	-0.0002	0.884	
Singulisphaera	Control-200mg/kg	0.097	1.000	0.673	0.406	0.500
	Control-400mg/kg	0.0001	0.0008	1.591	0.308	
	200mg/kg-400mg/kg	0.216	1.000	0.883	0.714	
Pseudosulfitobacter	Control-200mg/kg	0.0001	0.034	-1.194	0.288	0.417
	Control-400mg/kg	0.541	1.000	-0.366	0.598	
	200mg/kg-400mg/kg	0.146	1.000	0.682	0.469	
Planktosalinus	Control-200mg/kg	0.0001	0.034	-1.461	0.354	0.167
	Control-400mg/kg	0.0001	0.004	-1.723	0.384	
	200mg/kg-400mg/kg	1.0000	1.0000	0	0.045	
Tuberibacillus	Control-200mg/kg	0.0001	0.114	-0.778	0.213	0.625
	Control-400mg/kg	0.655	1.000	0.338	0.757	
	200mg/kg-400mg/kg	0.0001	0.022	1.517	0.335	
Methylacidimicrobium	Control-200mg/kg	0.117	1.000	0.431	0.275	0.417
	Control-400mg/kg	0.0001	0.004	1.399	0.309	
	200mg/kg-400mg/kg	0.110	1.000	0.998	0.625	
Species						
GW2011_GWF2_39_15	Control-200mg/kg	0.025	1.000	1.171	0.524	0.458
	Control-400mg/kg	0.0001	0.005	1.716	0.379	
	200mg/kg-400mg/kg	0.662	1.000	0.325	0.744	
uncultured bacterium contig00053	Control-200mg/kg	0.0001	0.161	1.511	0.428	0.583
	Control-400mg/kg	0.0001	0.032	2.203	0.558	
	200mg/kg-400mg/kg	0.741	1.000	0.372	1.128	
Paenibacillus silvisoli	Control-200mg/kg	0.341	1.000	0.954	1.002	0.958

	Control-400mg/kg	0.0001	0.039	2.131	0.551	
	200mg/kg-400mg/kg	0.322	1.000	0.921	0.930	
Gracilibacillus salitolerans	Control-200mg/kg	0.006	0.632	2.229	0.817	0.875
	Control-400mg/kg	0.0001	0.042	2.503	0.653	
	200mg/kg-400mg/kg	0.855	1.000	0.147	0.803	
Luteolibacter arcticus	Control-200mg/kg	0.0001	0.129	2.158	0.590	0.750
	Control-400mg/kg	0.0001	0.027	2.015	0.503	
	200mg/kg-400mg/kg	0.618	1.000	0.423	0.849	
Candidatus *Marinamargulisbacteria* bacterium SCGC AG-410-N11	Control-200mg/kg	0.001	0.211	1.630	0.480	0.583
	Control-400mg/kg	0.0001	0.042	2.249	0.584	
	200mg/kg-400mg/kg	0.865	1.000	0.178	1.046	
Halalkalibacter urbisdiaboli	Control-200mg/kg	0.0001	0.009	2.228	0.494	0.708
	Control-400mg/kg	0.0001	0.0001	2.215	0.429	
	200mg/kg-400mg/kg	0.995	1.000	0.009	1.339	
Planococcus sp. MSAK28401	Control-200mg/kg	0.253	1.000	-0.709	0.621	0.542
	Control-400mg/kg	0.0001	0.023	-3.982	0.977	
	200mg/kg-400mg/kg	0.001	0.255	-3.200	0.948	
Methanobrevibacter wolinii	Control-200mg/kg	0.089	1.000	-1.183	0.695	0.667
	Control-400mg/kg	0.0001	0.014	-2.691	0.634	
	200mg/kg-400mg/kg	0.165	1.000	-1.039	0.749	
uncultured bacterium contig00077	Control-200mg/kg	0.0001	0.003	3.509	0.730	0.792
	Control-400mg/kg	0.0001	0.0001	3.935	0.541	
	200mg/kg-400mg/kg	0.921	1.000	0.087	0.879	
Terasakiella pusilla	Control-200mg/kg	0.0001	0.025	1.621	0.382	0.542
	Control-400mg/kg	0.386	1.000	0.508	0.586	
	200mg/kg-400mg/kg	NA	NA	-1.073	NA	
KO						
K02491	Control-200mg/kg	0.257	0.957	-1.119	0.988	0.333
	Control-400mg/kg	0.0001	0.020	-1.737	0.390	
	200mg/kg-400mg/kg	0.158	1.000	-0.511	0.361	
K25919	Control-200mg/kg	0.044	0.956	2.278	1.129	0.875
	Control-400mg/kg	0.0001	0.002	3.291	0.661	
	200mg/kg-400mg/kg	0.709	1.000	0.406	0.475	
K02155	Control-200mg/kg	0.034	0.957	1.728	0.816	0.542
	Control-400mg/kg	0.0001	0.0003	2.086	0.382	
	200mg/kg-400mg/kg	0.392	1.000	0.406	0.475	
K06977	Control-200mg/kg	0.0001	0.022	1.653	0.355	0.625
	Control-400mg/kg	0.0001	0.121	1.705	0.447	
	200mg/kg-400mg/kg	0.690	1.000	0.290	0.727	
eggNOG						
transcriptional regulators	Control-200mg/kg	0.288	1.000	0.594	0.559	0.500
	Control-400mg/kg	0.075	1.000	-0.775	0.436	
	200mg/kg-400mg/kg	0.0001	0.023	-1.698	0.354	

containing th(...)_19883						
Glucosamine-6-phosphate deaminase	Control-200mg/kg	0.0001	0.050	2.454	0.547	0.625
	Control-400mg/kg	0.0001	0.0001	3.017	0.358	
	200mg/kg-400mg/kg	0.845	1.000	0.201	1.031	
ER retention sequence binding	Control-200mg/kg	0.0001	0.050	1.848	0.411	0.500
	Control-400mg/kg	0.081	1.000	0.788	0.451	
	200mg/kg-400mg/kg	0.082	1.000	-0.889	0.511	
Probably involved in the insertion of Fe(...)_1211	Control-200mg/kg	0.0001	0.050	1.369	0.302	0.500
	Control-400mg/kg	0.001	0.669	1.192	0.363	
	200mg/kg-400mg/kg	0.695	1.000	0.125	0.319	
TIGRFAM RNA polymerase sigma factor RpoS	Control-200mg/kg	0.857	1.000	0.153	0.848	0.625
	Control-400mg/kg	0.0001	0.001	-1.777	0.337	
	200mg/kg-400mg/kg	0.0001	0.0001	-2.260	0.329	
Purine nucleoside phosphorylase DeoD-type	Control-200mg/kg	0.386	1.000	-0.507	0.585	0.458
	Control-400mg/kg	0.0001	0.0001	-2.172	0.388	
	200mg/kg-400mg/kg	0.014	1.000	-1.243	0.504	
Bacterial toxin homologue of phage lysozyme, C-term	Control-200mg/kg	0.0001	0.004	1.645	0.313	0.417
	Control-400mg/kg	0.059	1.000	0.832	0.440	
	200mg/kg-400mg/kg	0.809	1.000	0.142	0.585	
Nitrogen-fixing NifU domain protein	Control-200mg/kg	0.001	1.000	1.749	0.549	0.500
	Control-400mg/kg	0.0001	0.002	2.239	0.437	
	200mg/kg-400mg/kg	0.681	1.000	0.245	0.597	
Essential cell division protein that coo(...)_4183	Control-200mg/kg	0.034	1.000	0.758	0.357	0.500
	Control-400mg/kg	0.0001	0.006	1.298	0.267	
	200mg/kg-400mg/kg	0.423	1.000	0.396	0.495	

P, raw *P*-value; *Q*, FDR-adjusted *P*-value; log_2FC, log_2 fold-change representing effect size; Prevalence denotes the detection rate of each feature across all samples. Features were pre-filtered based on prevalence; only features detected in at least 15% of total samples were included for differential abundance testing. NA indicates that statistical metrics could not be calculated due to zero counts within the compared groups.
